# Low preventive treatment initiation and completion among young children contacts of active TB in Tbilisi, Georgia

**DOI:** 10.5588/ijtldopen.24.0594

**Published:** 2025-04-09

**Authors:** E. Kokhreidze, R.R. Kempker, N. Tukvadze, M.C. Schechter, Z. Avaliani, H.M. Blumberg, M. Butsashvili, N. Lomtadze

**Affiliations:** ^1^ European University, Tbilisi, Georgia;; ^2^ National Center for Tuberculosis and Lung Diseases, Tbilisi, Georgia;; ^3^ Division of Infectious Diseases, Department of Medicine, Emory University School of Medicine, Atlanta, Georgia, USA;; ^5^ University of Georgia, Tbilisi, Georgia.

**Keywords:** latent tuberculosis infection, isoniazid, preventive treatment, child contact investigation

## Abstract

**BACKGROUND:**

Within the Georgian National Tuberculosis Program (NTP), TB preventive treatment (TPT) is recommended for all child TB contacts ≤5 years. To assess adherence to this guidance, we evaluated the care cascade for the treatment of latent TB infection among children ≤5 years at the National Center for Tuberculosis and Lung Disease (NCTLD).

**METHODS:**

We performed a cohort study among children ≤5 years evaluated at NCTLD during 2012–2014 as a contact of active pulmonary TB people. We recorded how many patients were recommended to start isoniazid preventive therapy (IPT) and how many initiated and completed treatment.

**RESULTS:**

Among 172 contacts of active TB people who visited the NCTLD between 2012 and 2014, 134 (74%) were contacts to drug-susceptible TB index cases. Among children recommended to start IPT (*n*= 94), 50 (51%) initiated treatment, and 16 (33%) completed IPT. Overall, 4 (4%) contacts developed active TB during 1,483 person-years of follow-up (mean 8.6 years), including 1 in the IPT group who did not complete therapy and 3 (6%) in the non-IPT group.

**CONCLUSIONS:**

Our findings highlight low rates of LTBI treatment recommendation, TPT initiation and completion among young children who were TB contacts in Georgia, highlighting the need for improved monitoring and enhanced treatment programs and regimens.

A key component of the WHO End TB Strategy is to prevent TB disease through TB preventive treatment (TPT).^1^ Implementing this intervention is especially critical among young children who are at increased risk of progressing to active TB disease following infection with *Mycobacterium tuberculosis*.^2^ Historically, due to limited resources and inadequate tools, high TB burden countries in low- and middle-income countries have focused on diagnosing and treating people with active TB with a lower priority placed on detection and treatment of latent TB infection (LTBI). The momentum to combat LTBI has increased in recent years, partly driven by the goal outlined at the 2018 UN High-Level Meeting on TB to provide TPT to 30 million people by 2022.^3^ WHO indicated an increase in the number of people with HIV and household contacts of active TB patients treated with TPT from 1 million in 2015 to 3.6 million in 2019, but during the COVID-19 pandemic, this number fell again to 3.2 million people in 2020.^2^ The target of treating 30 million people for LTBI during 2018–2022 was not met, and more needs to be done to scale up TPT programmes.^4^

Children are an important and high-priority group to target for TPT. Children and adolescents account for 11% (∼1.1 million) of all people with active TB annually, with half occurring in those <5 years of age who are at much higher risk of progressing to active TB disease following infection with *M. tuberculosis*. Thus, providing TPT to children <5 years of age is critical in preventing disease in this vulnerable age group.^5^ Importantly, TPT has been shown to be highly effective and well tolerated in children; however, implementation has been challenging, with available data showing low adherence and treatment completion rates.^6^ Relative to adults, studies on the LTBI care cascade in children remain limited, and further data are needed to help identify the main barriers and challenges in initiating and completing TPT.^7^

The main aim of this study was to evaluate the LTBI care cascade among young children who were household contacts of active TB index cases in the country of Georgia. During the study period, children aged ≤5 years who were household contacts of active TB index cases were recommended to receive 6 months of isoniazid (INH) daily. An additional goal of our study was to identify gaps in the LTBI care cascade that could be addressed by future interventions to enhance TPT completion rates in Georgia and other low- and middle-income settings.

## METHODS

A retrospective cohort study design was utilized. Children <5 years of age who were household contacts of active TB index cases in Tbilisi, Georgia, who presented to the National Center for Tuberculosis and Lung Disease (NCTLD) from 2012 to 2014 were included and assessed for TPT recommendation, initiation and completion, and the development of active TB disease through 2020. During the study period (2012–2014 years), active and passive surveillance was utilized by the Georgian NTP among all patients with confirmed (acid-fast bacilli[AFB] sputum smear and/or culture-positive) pulmonary TB. For passive surveillance, after index patients were diagnosed with active TB, they were asked to list all close (defined by the WHO as a person who is not in the household but shared an enclosed space) and household contacts(defined by the WHO as a person who shared the same enclosed living space for one or more nights or frequent or extended periods during the day with the index case during the 3 months before commencement of the current treatment episode) and to bring them to a local TB facility for investigation.^8^ In parallel with active surveillance, epidemiologists from the Georgian National Center for Diseases Control and Public Health (NCDC) were notified about all confirmed pulmonary TB people. Subsequently, they made a home visit within 2 months of diagnosis to perform symptom screening among all household contacts. After home visits, all close contacts were recommended to visit a TB facility for further screening.^9^ For all contacts evaluated at theNCTLD, a questionnaire, examination, and chest radiography were performed to rule out active TB disease. For patients with suspected active TB disease, sputum was collected for smear microscopy and culture. Additionally, all household contacts who were children (≤5 years) had a tuberculin skin test (TST) administered and read with a positive result test result defined as ≥5mm in duration. Given the high bacille Calmette-Guérin (BCG) vaccination coverage in Georgia (94%), which can lead to positive TST results due to cross-reactivity, all children with a history of exposure to LTBI were recommended for TPT, irrespective of the level of TST induration.

During our study period, children (≤5 years) who were in close contact of drug-susceptible index TB people were recommended to receive a TPT regimen consisting of 6 months of daily INH after active TB disease was ruled out. Children who were contacts of MDR TB people were not recommended for TPT, given the lack of effective options available at the time. All children initiating TPT were scheduled monthly follow-up visits at the NCTLD to monitor treatment adherence and tolerability. INH pill was prescribed using weight-based dosing of 10mg/kg and given monthly to parents to provide daily to their children.

### Data collection

Medical charts of contacts ≤5 years of age were reviewed, and the following information was obtained via abstraction: TST result, chest X-ray results, treatment recommendation, treatment adherence and completion rates, and adverse events experienced during treatment. Data on TB index cases was collected from the Georgia National TB Programme database and included relationships to child contact, baseline sputum smear and culture results, drug susceptibility testing (DST) results, and clinical outcomes. Additionally, the Georgia National TB Program database was assessed on 14 February 2020 to determine if any of the child contacts had been diagnosed with active TB disease and had initiated active TB treatment.

The study was approved by the Ethics Committee (Institutional Review Board) of the National Center for Tuberculosis and Lung Diseases, Tbilisi, Georgia).

### Data analysis

All data were entered into an online REDCap (Vanderbilt University, Nashville, TN, USA) database;^10^ analysis was done using SPSS (SPSS, Armonk, NY, USA) and R v3.6.2 (R Computing, Vienna, Austria). Differences in categorical variables were tested using either a Fisher’s exact or χ^2^ test and for continuous variables, a Mann-Whitney or two-sample *t*-test was used as appropriate. Descriptive statistics were used to evaluate the IPT care cascade. We calculated the person’s follow-up time as the time between the baseline visit and the date the database was last checked (14 February 2020) or the date of active TB diagnosis, whichever occurred first.

## RESULTS

A total of 854 pulmonary TB people (AFB sputum smear and/or culture-positive) pulmonary cases were registered at the NCTLD from 2012 through 2014. Among these index patients, 119 (14%) reported having close contacts who were ≤5 years old, resulting in 177 young children contacts being referred and investigated. The rate of children under 5 in Georgia is 6.1%. Five (2.8%) contacts were diagnosed with prevalent active TB at the baseline visit and excluded from further analyses. Among the remaining 172 children <5 years of age who were contacts, 120 (69%) were household contacts, and 52 (31%) were non-household close contacts. Among these 172 children, 10 (6%) had a prior TST, including 3 with a previous positive TST and 2 who had previously initiated IPT. No data regarding IPT completion of previous treatment was available. A TST was performed at the baseline in 112 (65%) of the 172 children, and 65 (58%) had a positive result (indurate on >5 mm). Among the 169 children who had chest X-rays performed, none had abnormal results detected. As shown in the Table, most pulmonary index cases (96/119, 81%) had culture-confirmed TB and 23 (19%) had a positive sputum AFB smear with negative culture results. Among 172 contacts, 155 (90%) were contacts of a culture-positive index case, and 143 (83%) were contacts of an AFB smear-positive index case (with or without a paired positive culture).

**Table.** Characteristics of child contacts and the index TB case.

Characteristics	Total	INH received	INH not received	*P* value
	(*n*=172)	(*n*= 48)	(*n*= 124)	
	*n* (%)	*n* (%)	*n* (%)	
TPT recommended	94 (54)	48 (100)	46 (37)	<0.01
Male	105 (61)	24 (50)	81 (65)	0.08
Age, years, median [IQR]	2.7 [1.3–3.9]	2.5 [1.3–3.9]	2.7 [1.4–3.8]	0.90
Georgian ethnicity	156 (90)	43 (89)	113 (90)	1
TST done	112 (65)	34 (71)	78 (62)	0.37
TST-positive (*n* = 112)	65 (58)	20 (59)	45 (58)	1
Chest X-ray obtained	167 (97)	43 (90)	124 (99)	<.01
Index case characteristics				
Household contact	120 (70)	40 (83)	80 (64)	0.01
Smear-positive	143 (83)	45 (94)	98 (78)	0.02
Culture-positive	155 (90)	45 (94)	110 (88)	0.40
Culture and smear-positive	134 (77)	44 (92)	90 (72)	<.01
DS-TB	134 (77)	47 (98)	92 (70)	<.01
Previous treatment for active TB	27 (16)	4 (8)	23 (18)	0.15
Current TB episode outcome successful	135 (78)	43 (90)	92 (74)	0.02

INH = isoniazid; TPT = TB preventive treatment; IQR = interquartile range; TST = tuberculin skin test; DS-TB = drug-susceptible TB.

Among the 172 contacts who were children <5 years of age, 134 (74%) were exposed to index TB people with drug-susceptible TB people and 38 (26%) to a multidrug-resistant TB index case. The median age of the contacts was 2.7 years(IQR 1.3–3.9); 156 (90%) children were Georgian citizens, and 105 (61%) were male. The majority of children were tested for TST (*n* = 112, 65%); 65 (58%) had a positive result (>5mm). Among all index cases, 143 (83%) were smear-positive, 155 (90%) were culture-positive, and 134 (77%) were smear and culture-positive. 27 (16%) of TB index cases had been previously treated. 135 (78%) cases had a successful TB treatment outcome.

Among the 134 children exposed to a drug-susceptible index case, 94 (54%) were recommended to receive IPT. Subsequently, 48(52%) children initiated and 16 (9%) completed IPT. Among the 32 children not completing therapy, the mean duration of treatment prior to interruption was 10 weeks. Four (8%) of 48 children interrupted TPT due to adverse events, including nausea, loss of appetite and fatigue (Figure).

**Figure.**
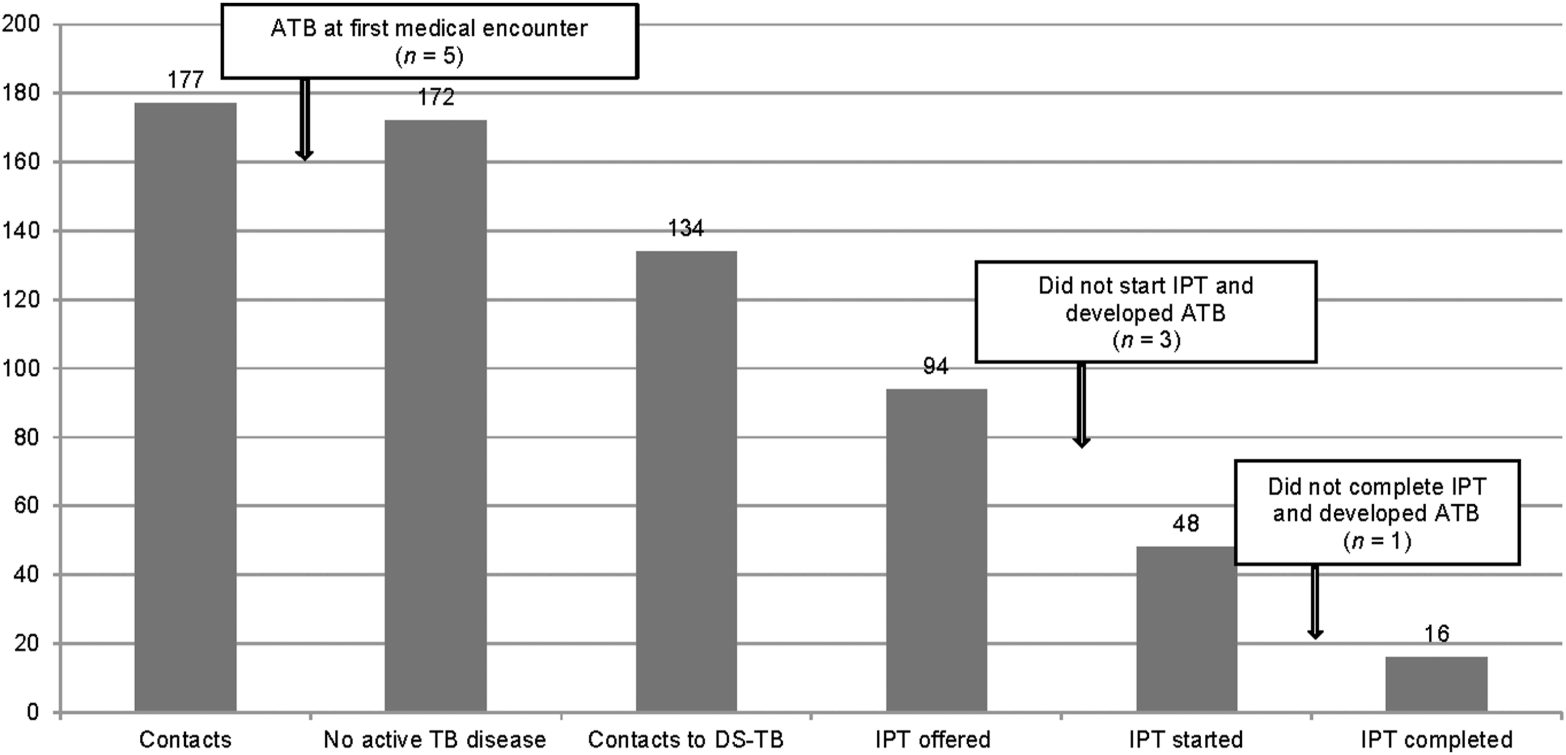
LTBI treatment care cascade among child contacts of active pulmonary TB people during 2012–2014. ATB = active TB; IPT = isoniazid preventive therapy; DS-TB = drug-susceptible TB; LTBI = latent TB infection.

During a total of 1,483 person-years follow-up time (mean 8.6 years per contact), 4 (2,3%) of 172 child contacts to a drug-susceptible and resistant case were diagnosed with active TB. The number of active TB diagnoses was 1 (2.1%) of 48 among those who started IPT and 3 (6.5%) of 46 among those who did not start IPT. The child who started IPT and developed active TB did not complete TPT (interrupted therapy after 3 months) and was diagnosed with bone TB 1 year after interruption. The median time between the baseline visit and TB diagnosis among those who did not start IPT was 90 days (range 13–532), and all had pulmonary disease. There were 0.27 TB people per 100 person-years in this cohort of young children who were contacts of active TB people.

## DISCUSSION

Our evaluation of the LTBI cascade of care among children ≤5 years revealed a low rate of TPT completion with a drop-off at every step of the care continuum. Per WHO guidelines, all children ≤5 years who are contacts of drug-susceptible pulmonary TB index cases should be treated for LTBI after active disease is ruled out, independent of TST results.^11^ However, our results indicated that only 12% (16/134) of this group completed IPT. This finding demonstrates the need to develop new approaches to implement such recommendations.^12^ Furthermore, our cascade of care findings demonstrate that there are multiple challenges to enhancing LTBI care, including physician recommendation for TPT, acceptance of treatment when offered, and completion of treatment after initiating. Additionally, in a country with high rates of drug-resistant TB, our data also highlights that there were no recommended treatments for LTBI among young children who were contacts of drug-resistant TB at the time and only limited options (fluoroquinolones) currently.^13^ Notably, the high rate of prevalent TB we found in child contacts (2.8%) demonstrates the value of contact tracing for active case-finding.

The results of a systematic review conducted by Martinez et al. between 1998 and 2018 found that 61% of TB development in children occurred within 90 days, which is close to our study results, with 3 of our four active TB diagnoses occurring within this timeframe.^5^ Also, it is vital to compare the period of contact investigation after confirmation of disease in the index case. Contact investigation time is meaningful. Our rate of TPT completion (9%) calls for urgent attention to developing ways to enhance LTBI treatment uptake and adherence. While our completion rate was the lowest we found in the literature, other reported completion rates from South Africa (15%), India (23%), Pakistan (33%), and Afghanistan (69%) were all less than optimal and indicate that strategies are needed in TB programs across the globe.^14^

One of the main challenges in implementing TPT is the duration of treatment. Updated guidelines from the WHO for shorter LTBI treatment regimens, including 12 weekly doses of INH and rifapentine (3HP) for a broader target group (contacts of DS- and DR-TB index cases in any age), may increase completion rates. The 3HP regimen was introduced into Georgian NTP guidelines in March 2020 and recommended for all contacts >2 years of DS-TB. Additionally, 6 months of daily levofloxacin was recommended for contacts, including children of MDR TB people, and importantly, this regimen is now supported by promising early released data from the TB-CHAMP study.^15^

### Limitations

Limitations of our study included its retrospective nature. The reasons for not recommending IPT and for treatment interruption were not wholly documented for many patients, preventing further detailed analysis. Furthermore, given that only children who presented to the NCTLD could be evaluated, we could not determine the total number of child contacts requiring TPT and developing active TB. In addition, exposures were from more than ten years ago. Despite these limitations, our study provides important insights into completion rates along each step of the LTBI cascade of care among children and extended follow-up for active TB disease.

## CONCLUSION

The LTBI cascade of care provides a helpful framework for identifying the challenges in enhancing TPT initiation and completion rates. Our results demonstrate that a multipronged approach is needed to enhance LTBI care among children and thus to enact a key component of the WHO END TB Strategy.
